# Circular RNA circPLK1 promotes breast cancer cell proliferation, migration and invasion by regulating miR-4500/IGF1 axis

**DOI:** 10.1186/s12935-020-01694-x

**Published:** 2020-12-09

**Authors:** Guanhong Lin, Shenyu Wang, Xinyu Zhang, Dan Wang

**Affiliations:** Department of Integrated Traditional Chinese and Western medicine, Liaoning Provincial Cancer Hospital, No.44 Xiaoheyan Road, Dadong District, Shenyang, 110000 Liaoning Province China

**Keywords:** Breast cancer, circPLK1, miR-4500, IGF1

## Abstract

**Background:**

Circular RNAs (circRNAs) can regulate gene expression in different malignancies. However, the biological functions of circRNA polo-like kinase-1 (circPLK1) in the tumorigenesis of breast cancer (BC) and its potential mechanisms have not been well elucidated yet.

**Methods:**

The expression levels of circPLK1, microRNA-4500 (miR-4500), insulin-like growth factor 1 (IGF1) were measured by quantitative real-time polymerase chain reaction (qRT-PCR) or western blot. Cell viability, cell cycle distribution, cell migration and invasion were determined by Cell Counting Kit-8 (CCK-8) assay, flow cytometry and transwell assay, respectively. Western blot assay was used to analyze the protein levels of cyclin-dependent kinase (CDK) 4 and CDK-6. The relationship between miR-4500 and circPLK1 or IGF1 was predicted by starBase v3.0 and verified by dual-luciferase reporter assay and RNA pull-down assay. The mice xenograft model was established to investigate the roles of circPLK1 *in vivo*.

**Results:**

CircPLK1 and IGF1 were upregulated and miR-4500 was downregulated in BC tissues and cells. Interference of circPLK1 inhibited BC cell growth, migration and invasion, which was reversed by overexpression of IGF1. Moreover, circPLK1 could directly bind to miR-4500 and IGF1 was verified as a direct target of miR-4500. Furthermore, IGF1 overexpression abated the inhibitory effects of miR-4500 upregulation on proliferation, migration and invasion of BC cells. Mechanically, circPLK1 was a sponge of miR-4500 to regulate IGF1 expression in BC cells. Besides, circPLK1 knockdown suppressed tumor growth via upregulating miR-4500 and downregulating IGF1.

**Conclusions:**

CircPLK1 silence inhibited BC cell growth, migration and invasion by regulating miR-4500/IGF1 axis, suggesting circPLK1/miR-4500/IGF axis might be a potential therapeutic target.

## Introduction

Breast cancer (BC) is the most commonly diagnosed cancer among females, and it is the leading cause of cancer death [1]. In 2018, about 2.1 million new cases of BC were diagnosed, accounting for a quarter of all cancer cases among women [2]. Although many treatments for BC, including radical surgery and adjuvant therapy, the prognosis for BC remains poor [3]. Hence, it is significant to better understand the molecular mechanisms of BC and develop more effective therapeutic strategies for treatment BC.

Circular RNAs (circRNAs) are a special type of non-coding RNAs (ncRNAs) that are widely expressed in mammals [4]. CircRNAs are characterized by covalently closed-loop structures with neither 5’ caps nor 3’ poly (A) tails, and circRNAs are conserved across species because of their resistance to RNase R (unlike lncRNA) [5]. Many reports have shown that circRNAs are involved in the regulation of gene transcription, suggesting that circRNAs play essential roles in multiple diseases, including cancer [6, 7]. Some circRNAs have been shown to play vital roles in regulation of BC progression [8–10]. Besides, a previous study indicated that circRNA polo-like kinase-1 (circPLK1; hsa_circ_0038632, chr16:23691404-23701688) was upregulated in triple-negative breast cancer (TNBC) and tightly related to poor survivals [11]. However, more roles and molecular mechanisms of circPLK1 in BC still need to be further explored.

Previous studies have indicated that circRNAs participate in many physiological and pathological processes by acting as competitive endogenous RNAs (ceRNAs) or microRNA (miRNA) sponges to regulate target genes and protein expression [12]. Hence, the circRNA-miRNA-mRNA axis may play a crucial role in the regulation of BC progression. A previous study showed that high level of insulin-like growth factor 1 (IGF1) was positively associated with bad prognosis in BC patients [13]. Moreover, miR-4500 has been identified to serve as an anti-oncogene in BC [14]. Bioinformatics tool predicted the putative binding sites between miR-4500 and circPLK1 or IGF1. Therefore, we supposed that circPLK1 might regulate BC progression through functioning as a sponge for miR-4500 to affect IGF1 expression.

In this work, we examined the expression of circPLK1, miR-4500 and IGF1 in BC tissues and cells, and explored their effects on BC cell growth, migration and invasion. Additionally, the potential regulatory mechanism among them in BC progression was also explored. The purpose of this research was to identify promising therapeutic targets for BC treatment.

## Materials and methods

### Tissue collection

In this research, BC tissues (n = 35) and adjacent non-tumor tissues (n = 35) were provided by the patients who had undergone surgery at Liaoning Provincial Cancer Hospital. All samples were timely frozen in liquid nitrogen and then maintained in −80 ℃. All enrolled patients signed the informed consents. The present study was approved by the Research Ethics Committee of Liaoning Provincial Cancer Hospital.

### Cell culture and transfection

Two BC cells (BT549 and HCC38) and breast epithelial cells (MCF-10A) were purchased from COBIOER (Nanjing, China). These cells were maintained in Dulbecco’s modified eagle medium (Invitrogen, Carlsbad, CA, USA) containing 10% fetal bovine serum (FBS, Invitrogen). Then these cells were cultivated at 37 ℃ under a humidified atmosphere with 5% CO_2_.

For transfection, short-hairpin RNA (shRNA) against circPLK1 (sh-circPLK1), circPLK1 overexpression vector (circPLK1), pcDNA3.0-IGF1 overexpression plasmid (pcDNA3.0-IGF1), miR-4500 mimic (miR-4500), miR-4500 inhibitor (anti-miR-4500), and negative controls (sh-NC, Vector, pcDNA3.0-NC, miR-NC, and anti-NC) were obtained from RiboBio (Guangzhou, China). Transient transfection was performed using the Lipofectamine 3000 reagent (Invitrogen).

### Quantitative real-time polymerase chain reaction (qRT-PCR)

Total RNA from tissues (tumor and normal) and cells (BT549, HCC38 and MCF-10A) was extracted using TRIzol reagent (Invitrogen). High-Capacity cDNA Reverse Transcription Kit and TaqMan MicroRNA Reverse Transcription Kit (Thermo Fisher Scientific, Waltham, MA, USA) were applied to synthesize complementary DNA (cDNA). Next, qRT-PCR was conducted in a 7500 Real-Time PCR System (Thermo Fisher Scientific) with PrimeScript RT Reagent Kit (Takara, Shiga, Japan). The sequences of primers used in this research were listed as followed: circPLK1 (Forward, 5′-ACTATGGTGGACAAGCTGCT-3′; Reverse, 5′-GGAGGGCAGCTATTAGGAGG-3′); PLK1 (Forward, 5′-GGCAACCTTTTCCTGAATGA-3′; Reverse, 5′-AATGGACCACACATCCACCT-3′); miR-4500 (Forward, 5′-TGAGGTAGTAGTTTCTTGCGCC-3′; Reverse, 5′-CTCTACAGCTATATTGCCAGCCAC-3′); IGF1 (Forward, 5′-GCTCTTCAGTTCGTGTGTGGA-3′; Reverse, 5′-CGACTGCTGGAGCCATACC-3′); glyceraldehyde-3-phosphate dehydrogenase (GAPDH) (Forward, 5′-GGCTGAGAACGGGAAGCTTGTCA-3′; Reverse, 5′-CGGCCATCACGCCACAGTTTC-3′); U6 (Forward, 5′-ATTGGAACGATACAGAGAAGATT-3′; Reverse, 5′-GGAACGCTTCACGAATTTG-3′). The expression of genes was normalized to GAPDH (for circPLK1, PLK1 and IGF1) or U6 (for miR-4500), and calculated with 2^−^^ΔΔCt^ method [15].

### RNase R treatment

RNase R (Epicentre Technologies, Madison, WI, USA) treatment was used to degrade linear RNA. In short, total RNA (2 µg) was incubated by RNase R (3 U/µg) for 0.5 h at 37 ℃. Next, the treated cells were harvested, and the corresponding levels were measured by qRT-PCR.

### Cell viability assay

Cell Counting Kit-8 (CCK-8; Boster, Wuhan, China) was employed to examine BC cell viability [16]. In brief, BT549 and HCC38 cells were seeded in 96-well plates and subjected to different treatments. After 48 h, CCK-8 solution (10 µL) was added to per well at 37 ℃ for 2–3 h. A microplate reader was utilized to examine the absorbance at 450 nm wavelength.

### Cell cycle assay

BT549 and HCC38 cells were seeded in 6-well plates and subjected to different treatments. After 48 h, BT549 and HCC38 cells were collected and fixed with ethanol (75%) overnight. Afterwards, cells were incubated with propidium iodide (PI; 25 µg/mL, Keygen, Nanjing, China), Triton X-100 (0.2%) and DNase-free RNase (20 µg/mL). After incubation for 15 min in the dark, flow cytometry (Partec AG, Arlesheim, Switzerland) was used for detecting cell cycle distribution.

### Western blot assay

Total protein was extracted using RIPA lysis buffer (Solarbio, Beijing, China). The protein concentration was evaluated by BCA protein assay kit (Solarbio), and then extracted protein samples (about 40 µg/lane) were loaded on sodium dodecyl sulfate-polyacrylamide gel electrophoresis (SDS-PAGE). The separated proteins were transferred onto polyvinylidene fluoride membranes, and then blocked with 5% nonfat milk for 1–2 h. These membranes were incubated by the primary antibodies (Abcam, Cambridge, UK), including cyclin-dependent kinase (CDK) 4 (1:2000, ab137675), CDK6 (1:2000, ab151247), IGF1 (1:1000, ab9572) and GAPDH (1:1000, ab37168). After that, membranes were then continuously incubated with secondary antibody (1:4000, ab205718, Abcam). After incubation for 2 h, immunoreactive bands were visualized by enhanced chemiluminescence reagent (Solarbio). ImageJ software was employed to assess the bands density [17].

### Transwell assay

For cell invasion assay, the upper chambers (Costar, Corning, NY, USA) were pre-coated by Matrigel (BD Biosciences, San Jose, CA, USA). Transfected cells (BT549 and HCC38) in 200 µL of serum-free medium (DMEM) were added to the top compartment of the chamber. At the same time, the bottom chambers were filled with complete medium (600 µL). Cells which had invaded into the lower chamber were then stained and photographed under a microscope at 100 × magnification. For cell migration, the steps were similar to cell invasion method except that the top chambers were not pre-coated with Matrigel.

### Dual-luciferase reporter assay

The potential complementary sequence of miR-4500 and circPLK1 or IGF1 was predicted using starBase v3.0. The wild-type sequences of circPLK1 or IGF1 containing the binding sites of miR-4500 were amplified and individually inserted into the pmirGlO luciferase reporter vector (Promega, Madison, WI, USA), thereby generating wild-type plasmids (wt-circPLK1 and wt-IGF1). Similarly, the mutant sequences were also designed and inserted into the pmirGLO vector to generate mutant-type plasmids (mut-circPLK1 and mut-IGF1). Briefly, the above reporters were co-transfected with miR-NC or miR-4500 into BT549 and HCC38 cells for 48 h. After that, dual-Luciferase Reporter Assay System (Promega) was employed for measuring the luciferase activity.

### RNA pull-down assay

RNA pull-down assay was carried out in BT549 and HCC38 cells using RNA-Protein Pull-Down Kit (Thermo Fisher Scientific). Briefly, miR-4500 and miR-NC were labeled with biotin and transfected into BT549 and HCC38 cells, respectively. Subsequently, streptavidin agarose beads were used to incubate cell lysates for 1 h. At last, qRT-PCR was employed for examining the levels of circPLK1 and IGF1.

### Xenograft tumor model

Female BALB/c nude mice (5–6 weeks old) were obtained from Huafukang (Beijing, China). Stably transfected HCC38 cells (sh-NC or sh-circPLK1) were injected subcutaneously into nude mice (n = 6/group). Tumor volume was detected at the indicated times and calculated as follows: volume = (length × width^2^)/2. After five weeks, mice were killed, and the formed tumors were excised, weighed and collected for further study. This *in vivo* experiments were approved by the Animal Care and Use Committee of Liaoning Provincial Cancer Hospital.

### Statistical analysis

All data from at least 3 independent experiments were presented as mean ± standard deviation (SD). GraphPad Prism was used for statistical analysis. Student’s *t*-test or a one-way analysis of variance (ANOVA) was applied to analyze significant differences between different groups. *P* value < 0.05 was considered as significant difference. ***P < 0.001.

## Results

### CircPLK1 was upregulated in BC tissues and cells

**Fig. 1:**
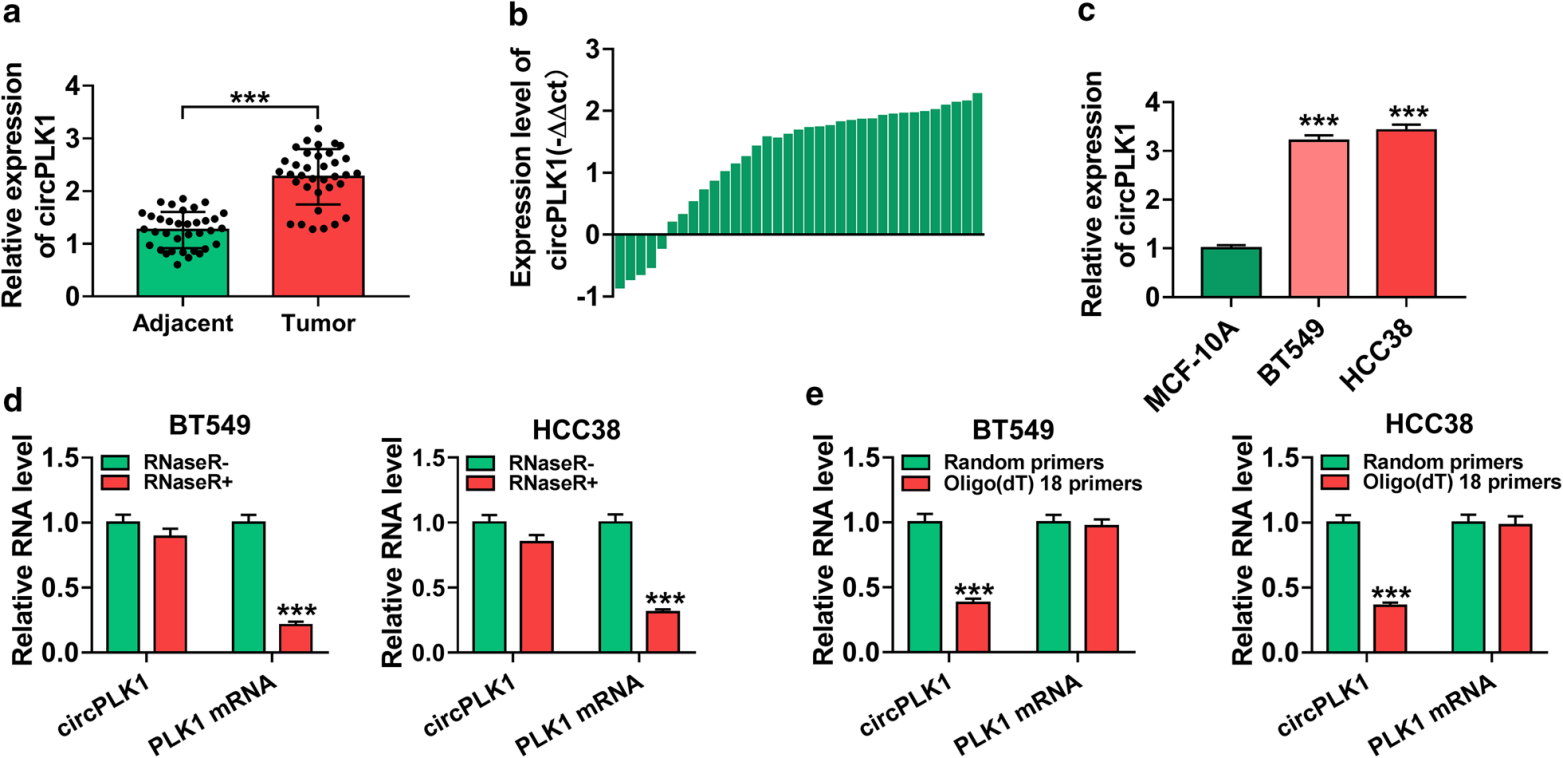
CircPLK1 expression was enhanced in BC tissues and cells. **a** The expression of circPLK1 was detected by qRT-PCR in 35 pairs of BC tissues and adjacent normal tissues. **b** ΔΔct is the difference between the ΔCT of BC tissues and the ΔCT of adjacent normal tissues. Relative circPLK1 expression in BC tissues (n = 35) compared with corresponding non-tumor tissues (n = 35). Positive − ΔΔct meant high circPLK1 expression. Negative − ΔΔct meant low circPLK1 expression. **c** CircPLK1 expression was determined by qRT-PCR in BC cells (BT549 and HCC38) and MCF-10A cells. **d** The relative levels of circPLK1 and PLK1 mRNA were measured after treatment with RNase R by qRT-PCR. **e** Relative RNA levels of circPLK1 and PLK1 mRNA were detected after reverse transcription with random primers and Oligo (dT) 18 primers by qRT-PCR. ***P < 0.001

To investigate the potential roles of circPLK1 in BC, its expression was determined in BC tissues and cells by qRT-PCR. As presented in Fig. 1a (*P* < 0.001), the expression of circPLK1 was increased in BC tissues (n = 35) compared with adjacent normal tissues (n = 35). To be specific, in 85.71% BC tissues (30 of 35), the expression of circPLK1 was upregulated compared to adjacent normal tissues (Fig. 1b). Likewise, circPLK1 expression was significantly higher in BC cells (BT549 and HCC38) than that in MCF-10A cells (Fig. 1c, *P* < 0.001). In general, RNase R can digest linear RNA but not circRNA. As illustrated in Fig. 1d (*P* < 0.001), PLK1 mRNA was markedly decreased after digestion by RNase R, but the expression of circPLK1 was not affected, implying the cyclic structure of circPLK1. Transcription assay was performed using random primers or Oligo (dT)18 primers. The results of qRT-PCR demonstrated that circPLK1 expression was strikingly lower when Oligo (dT)18 primers were used, than when random primers were used (Fig. 1e, *P* < 0.001), indicating that circPLK1 had no poly (A) tail. These results suggested the circPLK1 was upregulated in BC and had a closed-loop structure.

### **Knockdown of circPLK1 inhibited BC cell proliferation, migration and invasion**

.

**Fig. 2:**
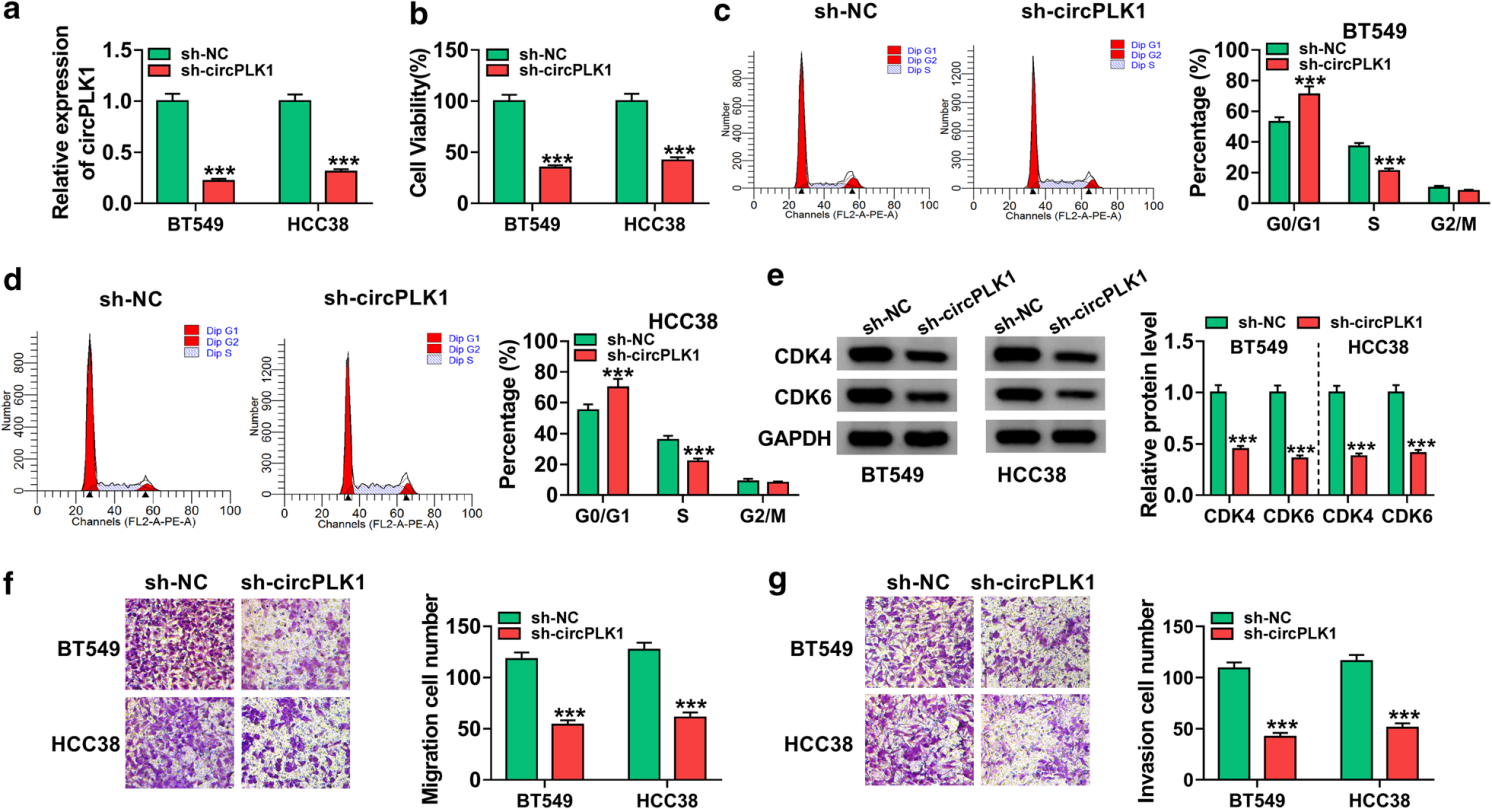
CircPLK1 knockdown limited BC cell growth, migration and invasion. BT549 and HCC38 cells were transfected with sh-NC or sh-circPLK1. **a** Knockdown efficiency of circPLK1 was evaluated by qRT-PCR. **b** CCK-8 assay was employed to evaluate cell viability. **c**, **d** Flow cytometry was applied to analyze the cell cycle distribution. **e** Western blot assay was conducted to examine the protein expression of CDK4 and CDK6. **f**, **g** Transwell assay was utilized to count the number of migrated and invaded cells (100 ×). ***P < 0.001

To explore the biological roles of circPLK1 in BC cells, BT549 and HCC38 cells were transfected with sh-NC or sh-circPLK1. Transfection efficiency of sh-circPLK1 was evaluated by qRT-PCR. The data showed that circPLK1 expression was conspicuously decreased in BT549 and HCC38 cells transfected with sh-circPLK1 compared to sh-NC group (Fig. 2a,* P* < 0.001). CCK-8 assay indicated that knockdown of circPLK1 greatly inhibited BT549 and HCC38 cell viability (Fig. 2b, *P* < 0.001). Cell cycle progression was assessed by flow cytometry. As presented in Fig. 2c and d (*P* < 0.001), interference of circPLK1 increased the percentage of G0/G1 phase cells and decreased the percentage of S phase cells in BT549 and HCC38 cells, suggesting that the cell cycle was arrested at the G0/G1 phase. CDK4 and CDK6 belong to D-type cyclins and regulate cell cycle transition from G1 to S phase to promote cell cycle progression [18]. Western blot assay suggested that the protein levels of CDK4 and CDK6 were reduced by knockdown of circPLK1 (Fig. 2e, *P *< 0.001). In addition, silence of circPLK1 inhibited migration and invasion of BT549 and HCC38 cells (Fig. 2f, g and* P* < 0.001). Our results indicated that circPLK1 might play a carcinogenic role in BC.

### IGF1 was overexpressed in BC tissues and cells

**Fig. 3:**
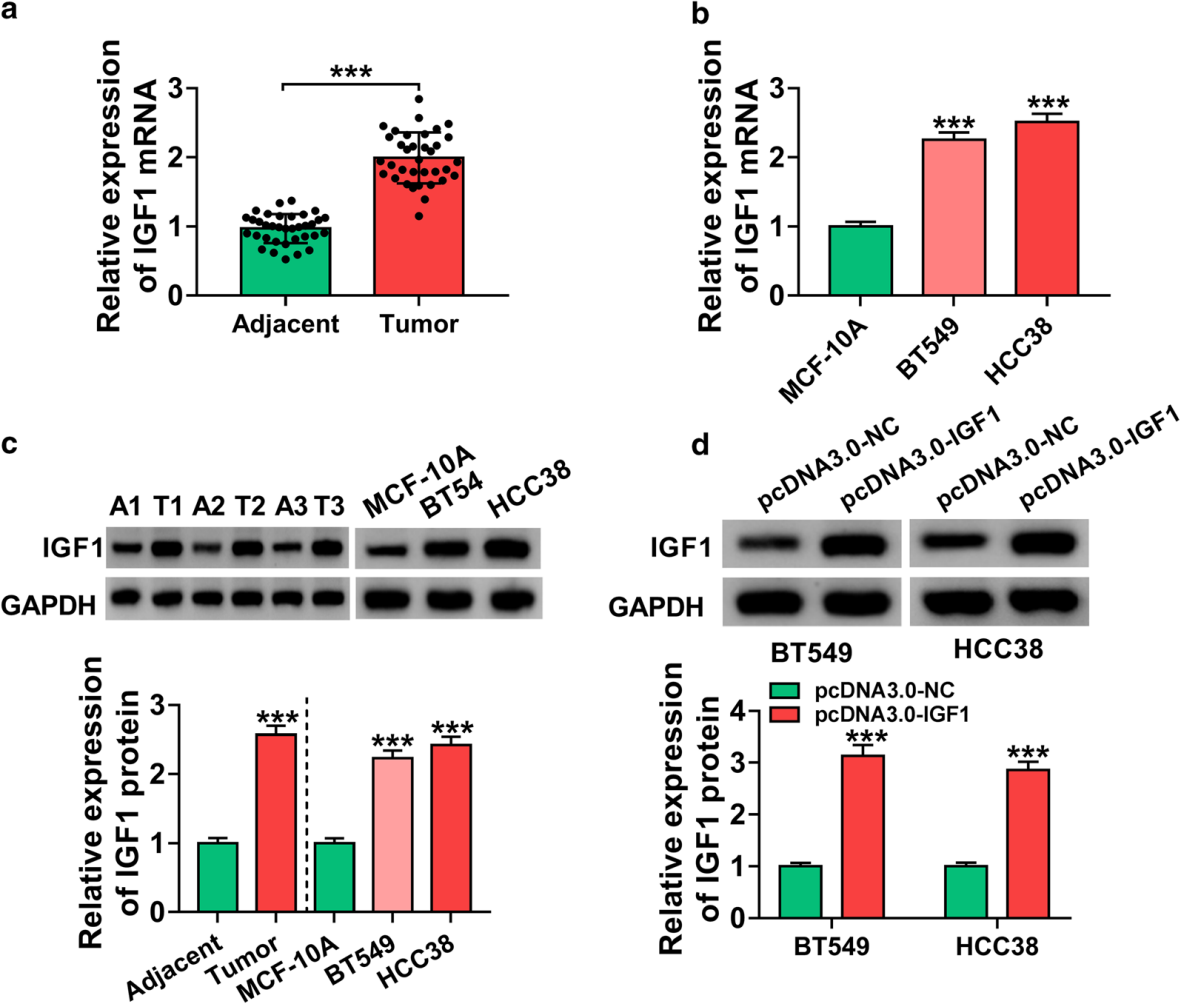
IGF1 was upregulated in BC tissues and cells. **a**–**c** IGF1 mRNA and protein levels were detected by qRT-PCR and western blot analyses in BC tissues, adjacent normal tissues, BC cells, and MCF-10A cells. **d** Overexpression efficiency of IGF1 was determined by western blot assay in BT549 and HCC38 cells transfected with pcDNA3.0-NC or pcDNA3.0-IGF1. ***P < 0.001

To explore the potential roles of IGF1 in BC, we analyzed the expression of IGF1 in BC tissues and cells. The results showed that IGF1 mRNA and protein levels were increased in BC tissues and cells compared with normal tissues and cells (Fig. 3a and c, *P* < 0.001). Moreover, overexpression efficiency of IGF1 was determined by western blot assay. After transfection with pcDNA3.0-IGF1, the protein expression of IGF1 was increased in BT549 and HCC38 cells (Fig. 3d, *P* < 0.001). These findings demonstrated that IGF1 might play a critical role in BC.

### IGF1 overexpression reversed the inhibitory effects of circPLK1 knockdown on proliferation, migration and invasion of BC cells

**Fig. 4:**
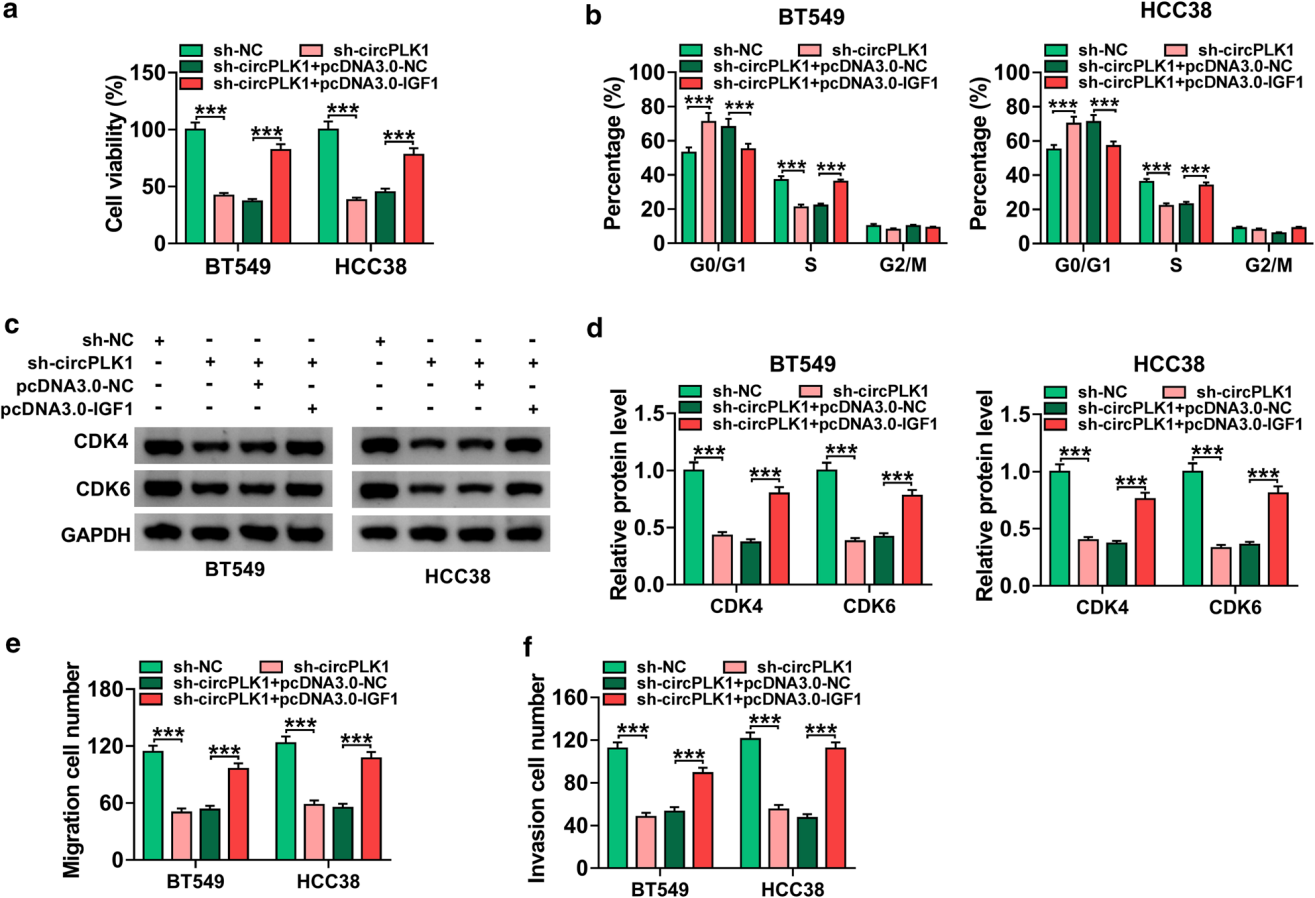
CircPLK1 silence inhibited BC cell growth, migration and invasion by downregulating IGF1. BT549 and HCC38 cells were transfected with sh-NC, sh-circPLK1, sh-circPLK1 + pcDNA3.0-NC, or sh-circPLK1 + pcDNA3.0-IGF1. **a** CCK-8 assay was employed to evaluate cell viability. **b** Cell cycle distribution was determined by flow cytometry. **c**, **d** The expression levels of CDK4 and CDK6 were analyzed by western blot analysis. **e**, **f** Cell migration and invasion were evaluated by transwell assay. ***P < 0.001

To investigate the relationship between circPLK1 and IGF1 in BC cells, BT549 and HCC38 cells were transfected with sh-NC, sh-circPLK1, sh-circPLK1 + pcDNA3.0-NC, or sh-circPLK1 + pcDNA3.0-IGF1. The suppressing effect of sh-circPLK1 on cell viability was abated by overexpression of IGF1 (Fig. 4a, *P* < 0.001). Moreover, upregulation of IGF1 reversed circPLK1 silence-mediated promotion of G0/G1 phase cells and reduction of S phase cells (Fig. 4b, *P* < 0.001). Furthermore, the decrease of CDK4 and CDK6 expression caused by circPLK1 knockdown was restored by transfection with pcDNA3.0-IGF1 (Fig. 4c, d and *P* < 0.001). Besides, overexpression of IGF1 also abolished the inhibitory effects of sh-circPLK1 on migration and invasion of BT549 and HCC38 cells (Fig. 4e, f and *P* < 0.001). Collectively, these data indicated that knockdown of circPLK1 inhibited proliferation, migration and invasion of BC cells by downregulating IGF1.

### MiR-4500 could bind to circPLK1 and IGF1 in BC cells

**Fig. 5:**
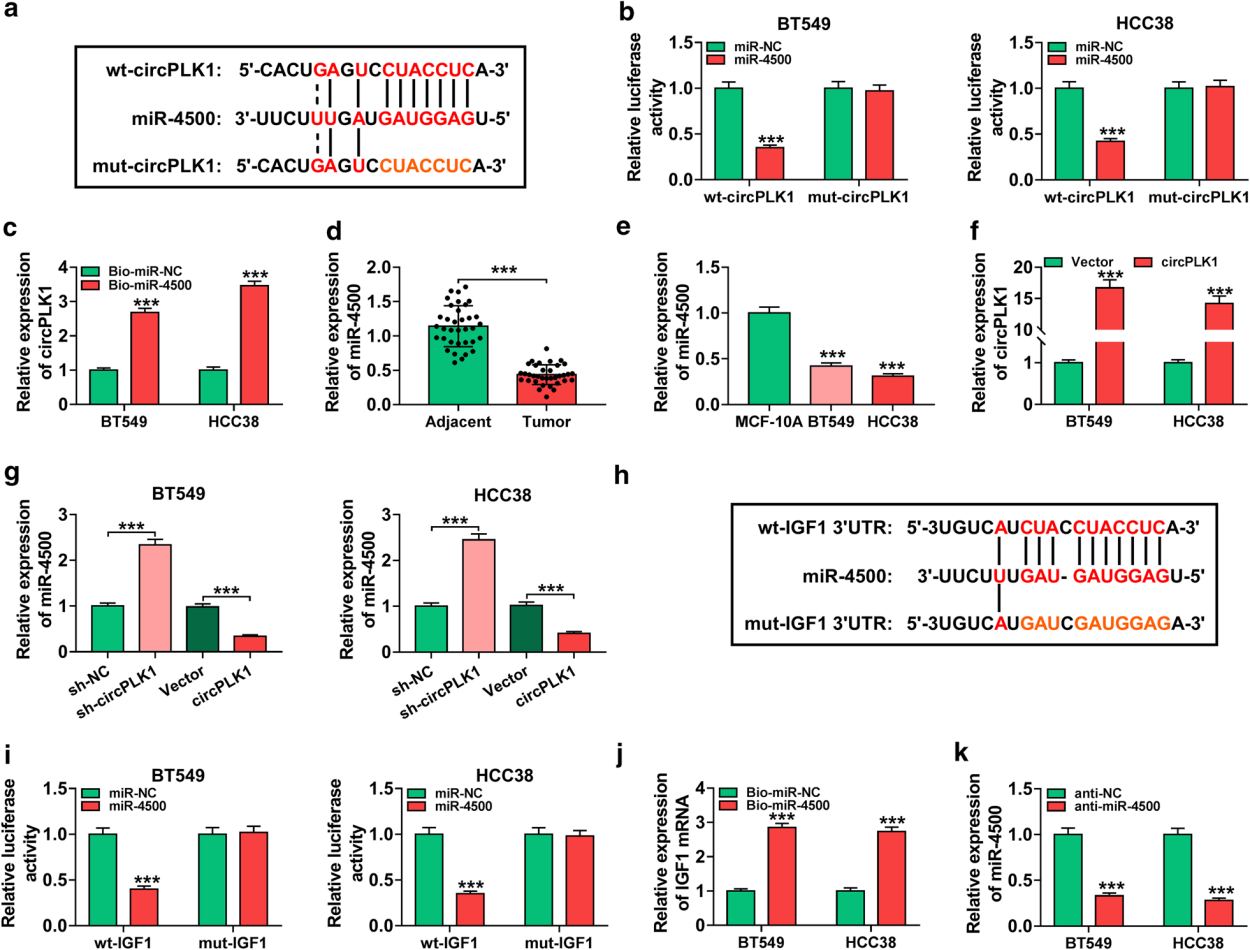
Interaction between miR-4500 and circPLK1 or IGF1 in BC cells. **a** StarBase v3.0 predicted the complementary sequences between circPLK1 and miR-4500. **b** BT549 and HCC38 cells were co-transfected with wt-circPLK1 or mut-circPLK1 and miR-NC or miR-4500, and then relative luciferase activity was measured. **c** Relative expression of circPLK1 was examined by RNA pull-down assay in BT549 and HCC38 cells incubated with Bio-miR-NC or Bio-miR-4500. **d**, **e** The expression of miR-4500 was examined by qRT-PCR and western blot analyses in BC tissues, adjacent normal tissues, BC cells, and MCF-10A cells. **f** Overexpression efficiency of circPLK1 was determined by qRT-PCR in BT549 and HCC38 cells transfected with Vector or circPLK1. **g** The level of miR-4500 was detected by qRT-PCR in BT549 and HCC38 cells transfected with sh-NC, sh-circPLK1, Vector, or circPLK1. **h** The putative binding sites between miR-4500 and IGF1 were predicted by starBase v3.0. **i** The luciferase activity in BT549 and HCC38 cells co-transfected wt-IGF1 or mut-IGF1 and miR-NC or miR-4500 was examined. **j** The mRNA expression of IGF1 was determined by RNA pull-down assay in BT549 and HCC38 cells incubated with Bio-miR-NC or Bio-miR-4500. **k** Knockdown efficiency of miR-4500 was determined by qRT-PCR in BT549 and HCC38 cells transfected with anti-NC or anti-miR-4500. ***P < 0.001

Previous studies suggested that circRNAs could serve as sponges for miRNAs to regulate target gene expression [6]. To explore whether circPLK1 acted as a sponge for miRNA, the potential targets of circPLK1 were predicted by starBase v3.0. The data showed that miR-4500 (a well-studied tumor suppressor) had putative binding sites for circPLK1 (Fig. 5a). Subsequently, dual-luciferase reporter assay and RNA pull-down assay were performed to confirm the prediction. The data showed that the luciferase activity of wt-circPLK1 was strikingly suppressed in BT549 and HCC38 cells transfected with miR-4500, while luciferase activity of mut-circPLK1 was not changed after transfection with miR-4500 (Fig. 5b, *P* < 0.001). Moreover, Bio-miR-4500 led to higher circPLK1 level than treatment of Bio-miR-NC in BT549 and HCC38 cells (Fig. 5c, *P* < 0.001). Next, the expression of miR-4500 in BC tissues and cells was measured. We found that miR-4500 level was reduced in BC tissues and cells relative to normal tissues and cells (Fig. 5d, e and *P* < 0.001). The expression of circPLK1 was increased in BT549 and HCC38 cells transfected with circPLK1 (Fig. 5f, *P* < 0.001), suggesting that circPLK1 was successfully transfected into BT549 and HCC38 cells. We further explored the influence of circPLK1 on miR-4500 expression. The results of qRT-PCR showed that knockdown of circPLK1 promoted miR-4500 expression, while overexpression of circPLK1 inhibited miR-4500 expression (Fig. 5g, *P* < 0.001), suggesting that circPLK1 negatively regulated miR-4500 expression. Interestingly, starBase v3.0 also showed that miR-4500 might bind to 3’UTR of IGF1 (Fig. 5h). To determine whether IGF1 directly interacted with miR-4500, dual-luciferase reporter assay and RNA pull-down assay were conducted. As shown in Fig. 5i (*P* < 0.001), overexpression of miR-4500 apparently repressed the luciferase activity of wt-IGF1 rather than the luciferase activity of mut-IGF1. Meanwhile, transfection of Bio-miR-4500 increased IGF1 mRNA expression, indicating interaction between miR-4500 and IGF1 (Fig. 5j, *P* < 0.001). Besides, the expression of miR-4500 was decreased after transfection with anti-miR-4500 (Fig. 5k, *P* < 0.001), implying that miR-4500 was successfully knocked down. These data suggested that miR-4500 was a target of circPLK1 and IGF1 was a target of miR-4500 in BC cells.

### Overexpression of miR-4500 suppressed BC cell proliferation, migration and invasion by downregulating IGF1

**Fig. 6:**
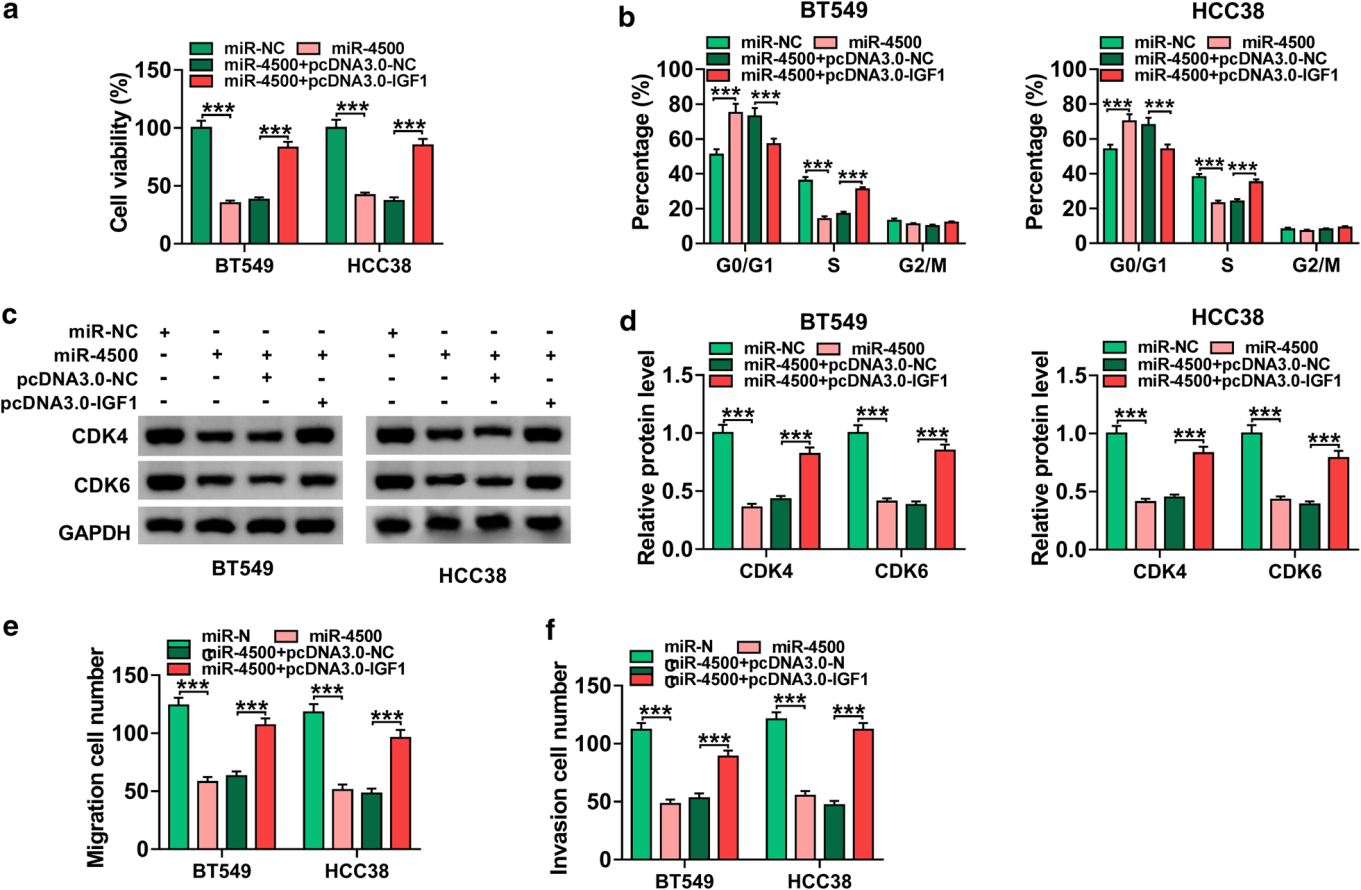
MiR-4500 exerted its anti-cancer effects in BC cells by targeting IGF1. BT549 and HCC38 cells were transfected with miR-NC, miR-4500, miR-4500 + pcDNA3.0-NC, or miR-4500 + pcDNA3.0-IGF1. **a** Cell viability was assessed by CCK-8 assay. **b** Flow cytometry was applied to analyze the cell cycle distribution. **c**, **d** Western blot assay was performed to measure the protein levels of CDK4 and CDK6. **e**, **f** Transwell assay was employed to determine cell migration and invasion. ***P < 0.001

To investigate whether IGF1 was involved in miR-4500-mediated functions in BC cells, BT549 and HCC38 cells were transfected with miR-NC, miR-4500, miR-4500 + pcDNA3.0-NC, or miR-4500 + pcDNA3.0-IGF1. Overexpression of miR-4500 inhibited cell viability as well as increased the percentage of G0/G1 phase cells and decreased the percentage of cells in S phase, which were abrogated following transfection with pcDNA3.0-IGF1 (Fig. 6a, b and *P* < 0.001). Moreover, the protein levels of CDK4 and CDK6 were reduced by upregulating miR-4500, which could be reversed by overexpression of IGF1 (Fig. 6c, d and *P* < 0.001). Meanwhile, upregulation of miR-4500 repressed migration and invasion of BT549 and HCC38 cells, while these effect were abated by upregulating IGF1 (Fig. 6e, f and *P* < 0.001). All these results demonstrated that miR-4500 exerted its biological functions by targeting IGF1 in BC cells.

### CircPLK1 positively regulated IGF1 expression by sponging miR-4500 in BC cells

**Fig. 7:**
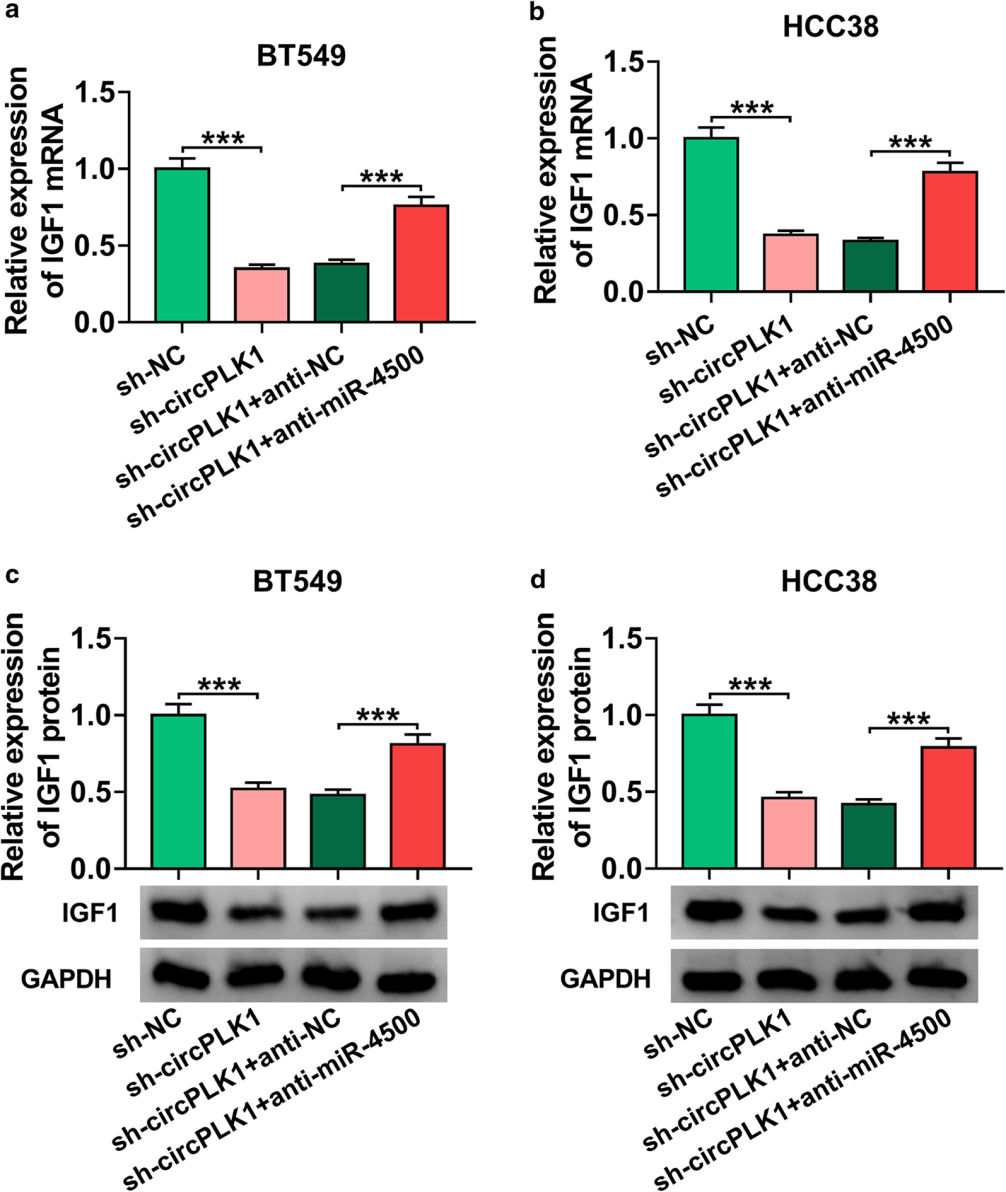
CircPLK1 regulated IGF1 expression through sponging miR-4500. **a**–**d** The mRNA and protein expression of IGF1 were measured by qRT-PCR and western blot analyses in BT549 and HCC38 cells transfected with sh-NC, sh-circPLK1, sh-circPLK1 + anti-NC, or sh-circPLK1 + anti-miR-4500. ***P < 0.001

To confirm whether circPLK1 acted as a molecular sponge of miR-4500 to regulate IGF1 expression, BT549 and HCC38 cells were transfected with sh-NC, sh-circPLK1, sh-circPLK1 + anti-NC, or sh-circPLK1 + anti-miR-4500. The results of qRT-PCR and western blot showed that silence of circPLK1 reduced the mRNA and protein abundance of IGF1, which was rescued by knockdown of miR-4500 (Fig. 7a and d, *P* < 0.001). Taken together, these findings demonstrated that circPLK1 served as a sponge of miR-4500 to regulate IGF1 expression.

### Interference of circPLK1 restrained tumor growth via regulating miR-4500 and IGF1 expression

**Fig. 8:**
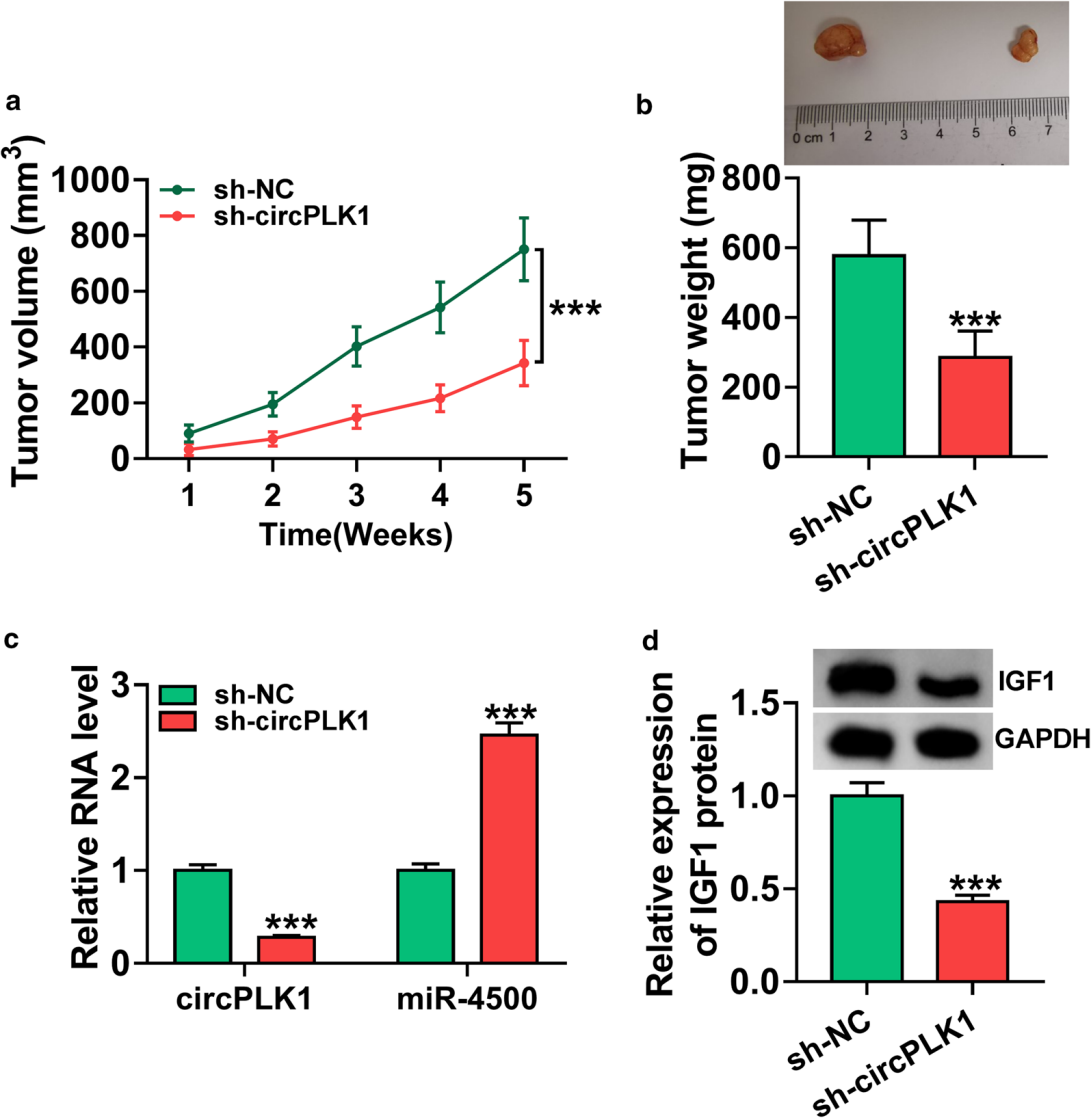
CircPLK1 knockdown repressed tumor growth via upregulating miR-4500 and downregulating IGF1. Sh-NC or sh-circPLK1-transfected HCC38 cells were introduced into nude mice to establish mice model. **a**, **b** The data of tumor volume and weight were recorded and graphed. **c** The expression levels of circPLK1 and miR-4500 were measured by qRT-PCR in excised tumor tissues. **d** The protein expression of IGF1 was determined by western blot assay in excised tumor tissues. ***P < 0.001

To verify the effect of circPLK1 on BC *in vivo*, sh-NC or sh-circPLK1-transfected HCC38 cells were introduced into nude mice. Knockdown of circPLK1 reduced tumor volume and weight in xenograft model (Fig. 8a, b and *P* < 0.001). The results of qRT-PCR proved that downregulation of circPLK1 inhibited the expression of circPLK1 and promoted the expression of miR-4500 in excised tumor masses (Fig. 8c, *P* < 0.001). Western blot analysis demonstrated that circPLK1 silence decreased the protein level of IGF1 in tumor tissues (Fig. 8d, *P* < 0.001). Altogether, these data showed that circPLK1 downregulation inhibited tumor growth by upregulating miR-4500 and downregulating IGF1.

## Discussion

BC is one of the most common and aggressive cancers among women, causing a large number of deaths [2]. Recent reports have demonstrated that circRNAs participate in a variety of cellular physiobiological processes and play vital roles in regulation of gene expression [19]. In the current research, we found that circPLK1 silence suppressed cell growth, migration and invasion by regulation of miR-4500/IGF1 pathway in BC cells.

Many reports have shown that circRNAs are considered as promising biomarkers for the diagnosis and prognosis of various cancers due to their abundance and stability in plasma and tissues [20]. Recent studies have confirmed that dysregulation of circRNAs is strongly linked to the occurrence and development of cancers, including BC. For example, Liang et al. demonstrated that circ-ABCB10 was upregulated in BC tissues, and its knockdown repressed the proliferation and accelerated cell apoptosis of BC cells [21]. Besides, Liu et al. revealed that hsa_circ_0008039 downregulation inhibited BC cell proliferation and migration via sponging miR-432-5p and inhibiting E2F3 expression [22]. More importantly, Kong et al. reported that circPLK1 was overexpressed in TNBC, and circPLK1 knockdown inhibited TNBC cell growth and invasion by regulating miR-296-5p/PLK1 axis [11]. In our research, high expression of circPLK1 was also observed in BC tissues and cells (Fig. 1), and its deficiency inhibited BC cell viability, arrested cell cycle progression, and repressed metastasis (Fig. 2). Likewise, circPLK1 knockdown led to the inhibition of tumor growth *in vivo* (Fig. 8). These findings disclosed that circPLK1 played a pivotal role in BC progression, and circPLK1 might be a promising prognostic biomarker and therapeutic target for BC.

IGF1, a tyrosine kinase receptor, has a significant influence on the control of cell and body size [23]. IGF1 has been acknowledged to be involved in diverse pathological processes, including cancer [24]. IGF1 was considered to be a key factor in the carcinogenesis of some tumors, including colon cancer [25], esophageal cancer [26], and lung cancer [27]. Notably, Walsh and his colleague pointed out that IGF1 increased invasive potential of MCF 7 breast cancer cells [28]. Nevertheless, the roles of IGF1 in BC cell growth and metastasis remain largely unknown. In this work, we uncovered that the expression of IGF1 was enhanced in BC tissues and cells (Fig. 3). Next, we explored whether IGF1 participated in circPLK1-mediated functions in BC cells. Rescue experiments showed that overexpression of IGF1 abated the suppressing effects of circPLK1 deficiency on cell growth, migration and invasion (Fig. 4). Our data proved that circPLK1 exerted its biological functions by regulating IGF1 expression.

An increasing number of studies have suggested that circRNA can serve as miRNA sponge to affect target gene expression [29, 30]. To explore the functional mechanism of circPLK1, the potential target miRNAs of circPLK1 were predicted by starBase v3.0. The data showed that miR-4500 might interact with circPLK1. Through dual-luciferase reporter and RNA pull-down assays, we validated the association between miR-4500 and circPLK1. Increasing evidence has confirmed that miRNAs exert their functional effects through binding to 3’UTR of target mRNAs [31]. So, potential target of miR-4500 was predicted by starBase v3.0. Interestingly, IGF1 was predicted as target for miR-4500. This prediction was confirmed by dual-luciferase reporter and RNA pull-down assays. Previous studies indicated that miR-4500 could inhibit the progression of many caners, including lung cancer [32], colorectal cancer [33], and papillary thyroid cancer [34]. Similarly, Li et al. proved that miR-4500 expression was declined in BC cells, and miR-4500 inhibited BC progression by downregulating RRM2 and inhibiting MAPK signaling pathway [14]. Consistent with this research, we also observed that miR-4500 was lowly expressed in BC (Fig. 5), and miR-4500 exerted anti-tumor roles via targeting IGF1 (Fig. 6). Besides, our study indicated that IGF1 expression was regulated by circPLK1/miR-4500 axis in BC (Fig. 7).

In conclusion, circPLK1 and IGF1 were highly expressed and miR-4500 was lowly expressed in BC. Moreover, our study for the first time proved that circPLK1 downregulation suppressed BC cell growth, migration and invasion via regulating miR-4500/IGF1 axis, which provided a new mechanism for better understanding the pathogenesis of BC.

## Data Availability

Please contact the correspondence author for the data request.

## Abbreviations

BC: Breast cancer
circRNAs: Circular RNAs
circPLK1: circRNA polo-like kinase-1
ceRNAs: Competitive endogenous RNAs
miRNA: microRNA
IGF1: Insulin-like growth factor 1
qRT-PCR: Quantitative real-time polymerase chain reaction
CCK-8: Cell Counting Kit-8
SDS-PAGE: Sodium dodecyl sulfate-polyacrylamide gel electrophoresis
CDK: Cyclin-dependent kinase
SD: Standard deviation
ANOVA: Analysis of variance
